# Reference Dependence in Bayesian Reasoning: Value Selection Bias, Congruence Effects, and Response Prompt Sensitivity

**DOI:** 10.3389/fpsyg.2022.729285

**Published:** 2022-03-17

**Authors:** Alaina Talboy, Sandra Schneider

**Affiliations:** ^1^Microsoft, Redmond, WA, United States; ^2^Department of Psychology, University of South Florida, Tampa, FL, United States

**Keywords:** reference dependence, problem presentation, problem solving, Bayesian reasoning, numeracy, PPV, problem structure

## Abstract

This work examines the influence of reference dependence, including value selection bias and congruence effects, on diagnostic reasoning. Across two studies, we explored how dependence on the initial problem structure influences the ability to solve simplified precursors to the more traditional Bayesian reasoning problems. Analyses evaluated accuracy and types of response errors as a function of congruence between the problem presentation and question of interest, amount of information, need for computation, and individual differences in numerical abilities. Across all problem variations, there was consistent and strong evidence of a value selection bias in that incorrect responses almost always conformed to values that were provided in the problem rather than other errors including those related to computation. The most consistent and unexpected error across all conditions in the first experiment was that people were often more likely to utilize the superordinate value (N) as part of their solution rather than the anticipated reference class values. This resulted in a weakened effect of congruence, with relatively low accuracy even in congruent conditions, and a dominant response error of the superordinate value. Experiment 2 confirmed that the introduction of a new sample drew attention away from the provided reference class, increasing reliance on the overall sample size. This superordinate preference error, along with the benefit of repeating the PPV reference class within the question, demonstrated the importance of reference dependence based on the salience of information within the response prompt. Throughout, higher numerical skills were generally associated with higher accuracy, whether calculations were required or not.

## Introduction

Diagnostic tests are used in many domains to help distinguish who has or does not have a condition of interest. However, these tests are not perfect. From an individual’s standpoint, knowing the likelihood that a positive test result indicates the presence of a condition is an important piece of information. This is the *positive predictive value* (PPV) of the test, which compares the subset of those who have the condition and test positive (C+T+) to all of those who test positive (T+).

These types of tests are commonly presented as Bayesian reasoning problems, which are used to evaluate the ability to update prior beliefs based on additional evidence to determine a posterior probability. Research over the last 40 years demonstrates that uninitiated or novice reasoners tend to have difficulty determining the PPV (e.g., Gigerenzer and Hoffrage, 1995; Gigerenzer et al., 2007; Reyna and Brainerd, 2008; Hoffrage et al., 2015; Johnson and Tubau, 2015, 2017; Sirota et al., 2015; Talboy and Schneider, 2017, 2018a,b).

The goal of the present research is to extend our previous efforts to identify factors that prevent reasoners from being ready to recognize and apply Bayes theorem to update probabilities based on diagnostic test information (e.g., Talboy and Schneider, 2018a,b). To do this, we deconstruct the problem into a simpler format that allows an assessment of whether and when reasoners can recognize and apply the needed information to infer the PPV. This decomposition into simplified problem forms is designed to get to the root of underlying difficulties in Bayesian reasoning. Our approach is to utilize a precursor to Bayesian reasoning that results in relatively high accuracy. Then, we systematically add higher order components of Bayesian reasoning problems to identify specific issues that reduce overall accuracy.

Factors associated with the low accuracy rates observed in Bayesian reasoning tasks are broadly categorized as either representational or computational difficulties (Johnson and Tubau, 2015, 2017; Talboy and Schneider, 2018a,b). Difficulties with how the problem is cognitively represented by reasoners are typically attributed to how the components of the problem relate to each other, which is more or less apparent depending on the formulation of the problem. For Bayesian reasoning problems, the greatest representational difficulty involves effectively communicating the nested structure of problems (Reyna and Brainerd, 2008; Johnson and Tubau, 2015) leading to what Johnson and Tubau (2017; Tubau et al., 2019) refer to as a relational alignment problem. Although many researchers have attempted manipulations to encourage reasoners to be aware of and understand this nested structure, accuracy typically falls short and is not consistent across manipulations (e.g., Brase, 2014; Sirota et al., 2014b,^2015^; Garcia-Retamero et al., 2015).

This representational issue may be compounded by the computational difficulties of extracting and computing the value needed to determine the PPV from the information provided in the problem (Gigerenzer and Hoffrage, 1995; Cosmides and Tooby, 1996; Macchi, 2000; Johnson and Tubau, 2017). The computational difficulties are especially apparent for those who struggle with numerical concepts compared to those who have stronger numerical skills (Schwartz et al., 1997; Reyna and Brainerd, 2008; Chapman and Liu, 2009; Johnson and Tubau, 2017; Talboy and Schneider, 2018b).

We posit that many of the representational and computational difficulties associated with Bayesian reasoning tasks are due to *reference dependence*, or the tendency to adopt a given or implied reference point at the start of cognitive deliberations. The contextual structuring provided by the problem description gives uninitiated reasoners a starting point from which to evaluate problem components, and may be perceived as providing signals of what is important in determining a solution (see, e.g., Hilton, 1995).

Across a variety of problem types, research suggests that inexperienced reasoners will often rely on the initial problem structure and organization to guide their approach to solution (Chi et al., 1981b; Talboy and Schneider, 2018a). Representational and computational difficulties are compounded in the standard Bayesian problem representation wherein the problem starts from one reference point (the condition) but the question asks reasoners to assess the information from another reference point (the test; Johnson and Tubau, 2013, 2017; Pighin et al., 2018; Talboy and Schneider, 2018a,b).

Relying on the given problem structure can be misleading when the structure presents information in a way that is not consistent with the question being asked (see also Gentner and Markman, 1997; Johnson and Tubau, 2015, 2017). We propose that this reference dependence plays a major role in the solutions that reasoners generate. We also posit that both reference dependence and difficulties discerning the structure of the problem contribute to what we refer to as a *value selection bias*, wherein problem solvers who are uncertain about the correct response opt for one of the values present in the problem rather than performing computations when they cannot readily discern how to reach an accurate solution (Gigerenzer et al., 1991; Gigerenzer and Hoffrage, 1995; Cosmides and Tooby, 1996; Galesic et al., 2009; Wolfe et al., 2013; Talboy and Schneider, 2017, 2018a). Similar biases have been observed, for instance, in conditional reasoning problems such as the Wason selection task (Wason, 1966; Evans and Lynch, 1973; Evans, 1998; Evans et al., 2003). When confronted with testing a conditional rule, reasoners tend to focus only on the values listed in the rule.

### Reference Dependence and Nested Problem Structures

One of the largest breakthroughs in improving Bayesian reasoning has come through the use of natural frequencies instead of single-event probabilities (e.g., Gigerenzer and Hoffrage, 1995; Gigerenzer et al., 2007; Garcia-Retamero and Hoffrage, 2013; McDowell and Jacobs, 2017). In this format, the PPV is calculated as a joint probability using a simplified form of Bayes Theorem rather than the more complex conditional probability algorithm (Gigerenzer and Hoffrage, 1995). This format boosts accuracy from about 10% to about 40% in the absence of training (e.g., Gigerenzer and Hoffrage, 1995; Micallef et al., 2012; Brase, 2014; Sirota et al., 2014a).

Despite this dramatic improvement with natural frequencies, well over half of participants across numerous studies still struggle to determine the correct solution to Bayesian reasoning problems. The primary difficulty seems to be identifying the correct reference class for determining the PPV. To find this requires understanding the nested structure of the problem as well as the subset-set relationships between the components of the problem (e.g., Barbey and Sloman, 2007; Sirota et al., 2014a; Girotto and Pighin, 2015; Brase and Hill, 2017).

Table 1 demonstrates how the nested values of a traditional Bayesian reasoning problem are organized using a contingency table (of case counts or frequencies). The sections presented with shading indicate which values are typically included in traditional presentations of Bayesian reasoning problems, such as the subset of those who have the condition and test positive (C+T+) and the subset of those who do not have the condition and test positive (C–T+). The unshaded boxes indicate values that could be deduced but are often not explicit, such as the complementary subsets of those who test negative (C+T– and C–T–).

**TABLE 1:** Bayesian reasoning task values organized in a 2 × 2 contingency table.

	Test positive	Test negative	*Marginal totals*
**Condition positive**	Condition positive and test positive (C+T+)	Condition positive and test negative (C+T–)	Total condition positive (C+)
**Condition negative**	Condition negative and test positive (C-T+)	Condition negative and test negative (C–T–)	Total condition negative (C–)
*Marginal totals*	Total test positive (T+)	Total test negative (T–)	Superordinate set (N)

The most critical of the absent values for arriving at PPV is the total of those who test positive (T+). This value is the reference class or denominator for determining PPV. Because the standard problem format focuses on condition base rates (C+ and C–), this condition-focused problem presentation is *incongruent* with the correct solution to the PPV question (Girotto and Gonzalez, 2001; Talboy and Schneider, 2017, 2018a,b; Pighin et al., 2018). Reasoners will often use the inappropriate condition-focus reference class as the denominator in their solution which yields the sensitivity of the test (C+T+ | C+) instead of the PPV (C+T+ | T+; Gigerenzer et al., 1991; Gigerenzer and Hoffrage, 1995; Cosmides and Tooby, 1996; Galesic et al., 2009; Wolfe et al., 2013; Talboy and Schneider, 2017, 2018a). The presence of a competing reference class total in the problem may cause a type of processing interference (e.g., Reyna, 2004; Reyna and Brainerd, 2008), which inhibits the reasoner’s ability to evaluate the problem from the alternate reference point.

We argue that many of the problems associated with the representation and computational components of Bayesian problem solving are tied to the cognitive process of reference dependence. Therefore, presenting the problem in a *congruent* format, wherein the T+ and T– reference classes are focal, should improve performance substantially. In previous studies, highlighting the T+ reference class boosted average accuracy to 80% or more (Girotto and Gonzalez, 2001; Talboy and Schneider, 2017, 2018a,b; Pighin et al., 2018).

Reference dependence is one of the most ubiquitous findings throughout the judgment and decision-making literature. A wealth of research indicates that decisions are highly dependent on the reference frame used to present choices (e.g., Tversky and Kahneman, 1991; Lopes and Oden, 1999; Hájek, 2007; Dinner et al., 2011; Jachimowicz et al., 2019), and that many decision heuristics, such as defaults and anchoring effects as well as framing effects, can be explained by reference dependence. Another example of reference dependence can be observed in the representativeness heuristic in which the prototype is adopted as the relevant reference class instead of taking the appropriate base rate into account (Kahneman et al., 1982; see also Gigerenzer and Murray, 1987). Although most research documenting reference dependence comes from the choice literature, the importance of context in shaping behavior has also been noted in other domains, including logical reasoning (Johnson-Laird, 2010), problem solving (Simon, 1973; Kotovsky and Simon, 1990), extensional reasoning (Fox and Levav, 2004)—and now in Bayesian reasoning as well (Talboy and Schneider, 2018a,b).

In what follows, we describe two studies investigating how problem presentation, and in particular, implied reference points, can influence naïve problem solvers when reasoning about the implications of diagnostic tests. To focus on the basic ability to identify the correct reference class, all of the problems are presented in a simplified frequency format, which transforms the Bayesian reasoning problem from one of conditional probability computations to a simpler reliance on joint probabilities.

## Experiment 1

The first experiment is described in three parts, although the data were collected contemporaneously. This provided the opportunity to explore how reference dependence may influence response accuracy in both congruent and incongruent problem-question pairings under a variety of conditions. For each pairing, we assessed how representational and computational difficulties interplay with the effect of reference class congruence on problem solving. We explored the relationship of these effects to individual differences in numerical skill and to error response patterns, with special attention to evidence of value selection bias versus computational errors.

Experiment 1a was designed to replicate the congruence effects observed in Talboy and Schneider (2018a) in the classic frequency-format partial subset problem and to explore whether these reference dependence effects generalize to problems with full subset information provided in the problem. Experiment 1b focuses on how the need for computation may complicate or enhance reference dependence effects, and Experiment 1c focuses on how the over-arching problem configuration into subset, superordinate, or no obvious reference classes impacts both accuracy and the type of response errors. Within each experiment, we also evaluated the relationship between numeracy and response accuracy to see if the previously observed relationship generalizes across problem formats (e.g., Talboy and Schneider, 2017, 2018a,b).

### Shared Methods

#### Participants

Three experiments were nested into a single large data collection and were run contemporaneously, with participants randomly assigned to one of 10 possible between-subjects conditions. Undergraduate volunteers participated for credit in psychology courses. Power analysis prior to data collection indicated a minimum of 40 participants needed per cell to find medium-large simple effects (η^2^ = 0.16; Cohen, 1992) with a power = 0.80 and α = 0.05 (which provided adequate power to identify smaller main effects, η^2^ = 0.06). After removing incomplete data from 5 participants, data from 589 participants (66% female; *n* = 58–59 per condition) were included for analyses. This research was approved by the University of South Florida’s Institutional Review Board.

#### Stimuli and Measures

Participants viewed problems in either an incongruent condition-focus or congruent test-focus format. An example of the differences in formats is provided in Table 2.

**TABLE 2:** Example condition-focus and test-focus presentations of the mammography problem.

Condition-Focus (CF)	Condition-Focus (CF)
In this sample of 10,000 women, 100 have breast cancer.	In this sample of 10,000 women, 1,070 received a positive result on their mammogram.
Of the 100 women who have breast cancer:	Of the 1,070 women who received a positive result on their mammogram:
80 received a positive result on their mammogram.	80 have breast cancer.
Of the 9,900 women who do not have breast cancer:	Of the 8,930 women who received a negative result on there mammogram:
990 received a positive result on their mammogram.	20 have breast cancer.
Imagine another random sample of 10,000 women who had a mammogram.	Imagine another random sample of 10,000 women who had a mammogram.

*Examples show the classic presentation which provides only partial subset information.*

All reasoning problems started with a general preamble about the condition of interest, as well as the test used for detecting the condition. The preamble stated that the test is not always correct and that specific information regarding correct and incorrect results is provided in the remaining description of the problem. The rest of the problem information was manipulated to conform to either a condition-focus or a test-focus problem presentation. This was followed by the PPV question: “Of the women from this new sample who test positive, how many do you expect to have breast cancer?” Answers were given as an open response requiring the correct identification of two relevant integer values (___ out of ___ people) in the correct order.

Eight Bayesian reasoning problems from previous research (Talboy and Schneider, 2018a), summarized in Table 3, were used to test participants’ abilities to understand and calculate the PPV. Specific conditions are introduced within the descriptions of Experiments 1a, 1b, and 1c below.

**TABLE 3:** Bayesian reasoning problems and relevant values.

		Base rate		True positive rate	False positive rate	PPV
Domain	Topic	C+	N	(C+T+) | (C+)	(C–T+) | (C–)	%
Medical	Mammogram	100	10,000	80 | 100	990 | 9,900	8
Medical	Diabetes	50	10,000	48 | 50	4,975 | 9,950	1
Legal	Polygraph	50	1,000	47 | 50	47 | 950	50
Legal	Recidivism	156	1,000	130 | 156	220 | 844	37
Sports	Baseball	185	250	130 | 185	15 | 65	90
Sports	Tennis	2,800	10,000	2,000 | 2,800	1,100 | 7,200	65
College	Employment	140	200	70 | 140	10 | 60	88
College	Exam Prep	350	500	275 | 350	25 | 150	92

*Base rate, the number of condition occurrences (C+) within the specific sample size (N). PPV, positive predictive value (% who correctly test positive out of all those who test positive. (C-T+| C–), the number of people who test positive (erroneously) out of the number of people who are actually negative. PPV is rounded to the nearest whole percentage.*

The dependent variable for all conditions was the number of correct PPV frequency responses across the eight Bayesian reasoning problems (range: 0–8 correct responses on both numerator and denominator response components). Because minimal or no calculations were needed to give the correct frequency responses, only exact values were coded as correct.

All participants also completed the Abbreviated Numeracy Scale (ANS; Weller et al., 2013). Numeracy is the ability to work with and understand numbers in various numeric formats (Peters et al., 2006, 2007). The ANS results in normally distributed scores and has generally demonstrated sufficient reliability and validity (Cronbach’s α = 0.71; Weller et al., 2013). In our sample of 589 participants, reliability was slightly lower (Cronbach’s α = 0.64; see also Talboy and Schneider, 2017, 2018a).

In preparation for analyses with ANS score as covariate, a check of covariance assumptions confirmed that numeracy was not systematically different across the 10 randomly assigned conditions, *F*(9,579) = 1.41, *p* = 0.18. The mean ANS score for Experiment 1 was 4.41 out of 8 (*SD* = 1.80, Min = 0, Max = 8). PPV accuracy means are adjusted throughout to hold ANS constant for comparisons (leading to slight differences in adjusted means across analyses).

#### Procedure

All data were collected during 1-h supervised sessions in a university computer lab equipped with 11 desktop computers. General instructions were read to participants, with additional instructions provided via computer. Participants first completed the Abbreviated Numeracy Scale. Then, an experimenter provided participants with a pencil and a blank paper form numbered 1 through 8 to record any notes they felt they needed to complete the next section.

Each participant completed the eight problems as randomly ordered in Qualtrics.com (algorithm from Matsumoto and Nishimura, 1998). Each problem was presented by itself with the frequency response format question. After completing each of the eight problems, participants were dismissed from the study.

## Experiment 1a: Partial Versus Full Information

Untrained reasoners tend to create mental representations of a problem based, often exclusively, on the information that is provided (Kintsch and Greeno, 1985; Johnson-Laird, 1994; Sirota et al., 2014a). In addition to congruence and value selection effects, this dependence on the given problem structure may also impede the creation of an accurate mental model when key information is missing. Findings most often demonstrate this reliance and show that reasoners tend to be insensitive to information that is intentionally left out of problems (Hammerton, 1973; Fischhoff et al., 1978; McDowell et al., 2016; McDowell and Jacobs, 2017). In some cases, however, reasoners (when asked) may make simple assumptions about information that they believe should be present but is not (e.g., Hamm et al., 1988). Because uninitiated reasoners tend to rely on surface features provided in the problem to guide how they determine the solution (Winner et al., 1980; Chi et al., 1981a,b; Owen and Sweller, 1989; Swanson and Beebe-Frankenberger, 2004), their responses may typically conform to surface features made available in the problem.

By providing a verbal presentation that includes all of the subsets from the contingency matrix, we hypothesize that reasoners may be more likely to develop a more complete mental model of the problem. This could help them understand the nature of the problem, which should help them resist the use of inappropriate reference points and find the path to solution, reducing the tendency to simply select given values for their responses when simple computations are needed for solution.

With partial presentations, implicit information may be missed or ignored (Hammerton, 1973; Fischhoff et al., 1978; McDowell et al., 2016; McDowell and Jacobs, 2017) or it may be encoded into the mental representation of the problem but in a way that is not immediately accessible for problem solving (Johnson-Laird, 1994). Given this, accuracy is expected to increase on problems with full subset compared to partial subset information (Johnson-Laird, 1994; Legrenzi and Girotto, 1995; Girotto and Gonzalez, 2001; Markovits and Barrouillet, 2002). By having all of the components available, reasoners do not need to mentally manage as many pieces of information, making it less likely for reasoning errors to occur (e.g., Markovits and Barrouillet, 2002).

However, there is a competing hypothesis. Including full subset information could decrease accuracy because reasoners need to discriminate among more values than with partial subset information. In choice tasks, having several options can be overwhelming, placing a larger cognitive burden on reasoners (Iyengar and Lepper, 2000; Greifeneder et al., 2010; Johnson et al., 2012). Reasoners may also get confused about which pieces of information are necessary to include in the mental model or to use for solution when they must discriminate among a larger set of values.

Experiment 1a tested whether providing complete subset information would facilitate, or perhaps hinder, the appropriate representation of incongruent and congruent problems.

### Method

#### Participants

Experiment 1a included 236 participants randomly assigned to one of four between-subjects conditions.

#### Design

Experiment 1a used a 2 × 2 Congruence (congruent, incongruent) × Information (partial subsets, full subsets) between-subjects design. Incongruent condition-focus problems involved a mismatch in the reference class provided in the problem and the test-focus needed for the PPV solution, whereas congruent test-focus problems matched in their emphasis on the test. Conditions with partial subset information, as in typical Bayesian problem presentations, only presented two of four subsets (as shown in Table 2). For the condition-focus problems, partial information included the condition base rate (C+ out of N), along with the C+T+ and C–T+ subsets. For the test-focus problems, the base rate of testing positive within a random sample was given (T+ out of N), along with the C+T+, and C+T– subsets. For full information conditions, all four subsets (C+T+, C+T–, C–T+, and C–T–) were included nested within the appropriate reference class. An example of the congruent and incongruent problem with full subset information is shown in Table 4.

**TABLE 4:** Example presentations with full subset information for the mammography problem.

Incongruent condition-Focus problem – Full subset information
To determine whether a woman is at risk of breast cancer, doctors conduct mammogram screenings. Sometimes women test positive even when they should test negative or test negative when they should test positive Here is some information for a random sample of 10,000 women who had a mammogram:
In this sample of 10,000 women, 100 have breast cancer.
Of the 100 women who have breast cancer:
80 received a positive result on their mammogram.
20 received a negative result on their mammogram.
Of the 9,900 women who do not have breast cancer:
990 received a positive result on their mammogram.
8,910 received a negative result on their mammogram.
Imagine another random sample of 10,000 women who had a mammogram.

**Congruent test-Focus problem – Full subset information**

To determine whether a woman is at risk of breast cancer, doctors conduct mammograms screenings. Sometimes women test positive even when they should test negative or test negative when they should test positive. Here is some information for a random sample of 10,000 women who had a mammogram:
In this sample of 10,000 women, 1,070 received a positive result on their mammogram.
Of the 1,070 women who received a positive result on their mammogram:
80 have breast cancer.
990 do not have breast cancer.
Of the 8,930 women who received a negative result on their mammogram:
20 have breast cancer.
8,910 do not have breast cancer.
Imagine another random sample of 10,000 women who had a mammogram.

### Results

A 2 × 2 Congruence (congruent, incongruent) × Information (partial subsets, full subsets) analysis of covariance [corroborated by Generalized Estimating Equations (GEE) repeated measures binary regression] was used to evaluate the effects of the two primary between-subjects variables on accuracy while controlling for differences in numeracy.

#### Numeracy

Consistent with previous findings (e.g., Peters et al., 2006; Garcia-Retamero and Galesic, 2010; Johnson and Tubau, 2017; Talboy and Schneider, 2017), numeracy significantly predicted PPV performance, *F*(1,231) = 55.19, *p* < 0.001, $\eta_{\text{p}}^{2}$ = 0.19 {Wald *C*^2^(1, *N* = 236) = 48.18, *p* < 0.001, Exp(B) = 1.59, 95% CI [1.39–1.81]}. Stronger numerical skills generally corresponded to higher accuracy rates on the Bayesian reasoning tasks, *r*_*s*_(234) = 0.45, *p* < 0.001.

#### Congruence and Amount of Information

As predicted, those who read congruent problem-question pairings (47%; *M*_*adj*_ = 3.73, *SD* = 2.79) were more accurate than those who read incongruent problem-question pairings (33%; *M*_*adj*_ = 2.61, *SD* = 2.79), *F*(1,231) = 9.38, *p* = 0.002, $\eta_{\text{p}}^{2}$ = 0.04 [Wald *C*^2^(1, *N* = 236) = 9.29, *p* < 0.002, OR = 1.96; 95% CI: 1.27, 3.03]. Nevertheless, the accuracy advantage for congruent problems over incongruent problems was not as large as observed in previous studies (Talboy and Schneider, 2018a,b). Thus, the main effect of congruence provides only modest support for the congruence hypothesis, which argues that the starting point provided by the problem presentation influences the reasoner’s ability to determine the solution.

**FIGURE 1:**
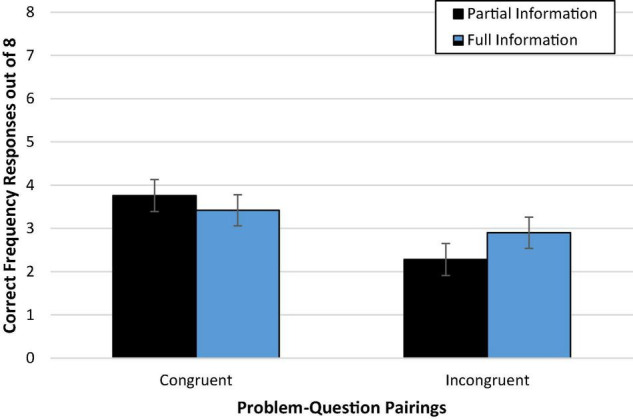
Experiment 1a condition accuracy means while controlling for numeracy. Error bars represent ± 1 SE.

There was no discernable difference in accuracy between those who read problems presented with partial information (39%; *M*_*adj*_ = 3.10, *SD* = 2.79) compared to full information (41%; *M*_*adj*_ = 3.24, *SD* = 2.79), *F* < 1 [Wald *C*^2^(1, N = 236) = 0.14, *ns*]. Additionally, Figure 1 demonstrates that there is little evidence of an interaction effect of congruence and amount of information on accuracy, *F*(1,231) = 1.53, *p* = 0.22 [Wald *C*^2^(1, *N* = 236) = 1.51, *ns*]. Against expectations, we did not find convincing support for either the mental models or the discrimination hypothesis. An exploratory *post hoc* comparison of condition means with Bonferroni correction suggested evidence closer to the mental models hypothesis in that congruence seemed to facilitate performance (versus incongruence) only with partial information (*p* = 0.016, *d* = 0.58), suggesting that, if anything, full information may reduce the need for the congruent problem formatting.

#### Response Error Patterns

We also examined the choice of reference class by analyzing denominator response patterns for each participant across the eight Bayesian reasoning problems to determine if any particular problem-relevant but incorrect value was routinely indicated (e.g., C+, N). Consistent with the congruence hypothesis, previous research has shown that reasoners often utilize the reference values provided in the problem presentation as part of their response, even when calculations are required (Talboy and Schneider, 2017, 2018a). Therefore, most denominator responses were expected to correctly conform to the T+ reference class in the congruent pairings but would tend toward the C+ reference class in the incongruent pairings, with a smaller portion of reasoners using other reference values provided in the problem such as N (i.e., the total sample).

**FIGURE 2:**
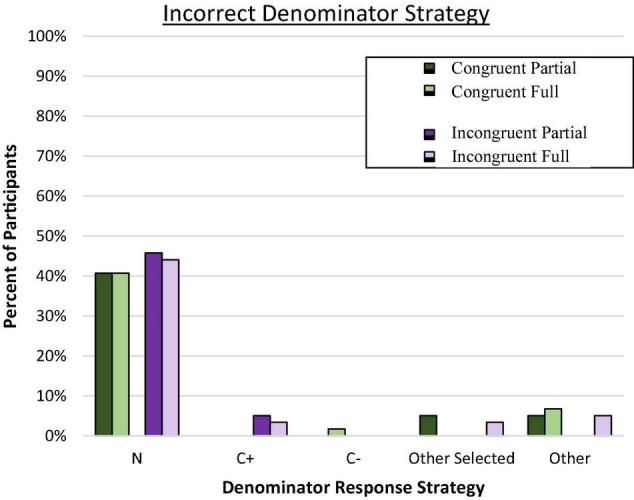
Proportion of participants who consistently used the incorrect denominator strategy on frequency responses. C± denotes the total number of people who have or do not have the condition. Total N denotes the total in the superordinate set (i.e., sample size).

Predominant response strategy for each participant was defined as four or more responses that conformed to the same problem value. “Other Selected” indicates those with inconsistent responses that predominantly came from the problem. The remainder were coded as “Other.” Although we expected substantial reliance on the focal reference class from the problem, a different pattern of response errors emerged as shown in Figure 2.

Evaluation of the error responses confirmed that the predominant incorrect strategy across all conditions involved selecting the overall sample size (N) rather than the conflicting reference class or any other incorrect value (*p* < 0.001 by binomial *z* in each condition). Moreover, the tendency to rely on the superordinate value (N) as the reference class rather than any other value (correct or incorrect) was similar across all four groups, *C*^2^(3, *N* = 236) = 0.47, *ns*. Very few participants (and then exclusively in incongruent conditions) consistently utilized the C+ reference class. In retrospect, we speculated that we may have inadvertently introduced a new, potentially dominant reference class by asking people to “imagine another random sample of 10,000 people…” which is the superordinate set (N). This may have nudged people toward selecting this salient value as the reference denominator, consistent with the proposed value selection bias. Reasoners in all conditions overwhelmingly utilized a value from the problem for their denominator responses.

### Discussion

In Experiment 1a, we replicated findings in favor of the numeracy, congruence, and value selection bias hypotheses, although the congruence effect was weak with disappointing accuracy levels. The inclusion of partial versus full subset information did not clearly affect accuracy, but full information may slightly reduce the benefit of congruent over incongruent pairings.

The substantially reduced effect of congruence overall could be the result of the addition of a response prompt adopted from examples in the literature (i.e., applying values to a new random sample; Gigerenzer and Hoffrage, 1995; Hoffrage and Gigerenzer, 1998). Introducing the instruction to imagine another sample could have suggested to participants that the superordinate value indicating sample size (N) should be viewed as the focal reference point (see also Johnson and Tubau, 2017). This change may have reduced any benefit or detriment for full subset over partial subset information. Nevertheless, the surprising finding suggests the potential importance of salient reference points as a primary determinant of answers to Bayesian reasoning problems.

## Experiment 1b: Selection Versus Calculation

The benefit of using a congruent problem-question pairing has been attributed to using reference dependence to ease the difficulties associated with both representation and computation of solutions (Talboy and Schneider, 2018a). With a congruent pairing, the organization of the problem information maps directly to the question of interest. This eliminates the need for problem re-structuring, but it also eliminates the need for computation, as the needed T+ denominator is provided as a value that can be selected directly from the problem. In contrast, the T+ denominator must be calculated in incongruent problem-question pairings from two component pieces in different reference classes (C+T+ and C–T+). Therefore, the requirements for solution in incongruent pairings is complicated by the additional need for computation.

Although some argue that these computations are rudimentary because they involve basic arithmetic operations like adding (Johnson-Laird et al., 1999; Sloman et al., 2003), there is substantial evidence demonstrating that reasoners often fail to complete these basic computations (Mayer, 2003; Reyna and Brainerd, 2008; Johnson and Tubau, 2015). There are at least two potential explanations for this failure. First, reasoners may be dependent on the problem structure, which leads them to utilize the reference class totals that are focused on in the problem, regardless of appropriateness for reaching the solution. Alternatively, reasoners may exhibit value selection bias, perhaps assuming that their task is to find the needed value from within the problem. Both of these possibilities are consistent with a bias toward cognitive ease (Kahneman, 2011), which favors a readily available answer over even seemingly innocuous arithmetic steps such as adding two values. As Ayal and Beyth-Marom (2014) have shown, accurate performance in solving probability-based reasoning problems drops off dramatically as the need for computations increases.

Whereas the partial versus full subset manipulation of Experiment 1a focused on a representational issue, it did not address differences in the need for computation. Experiment 1b was designed to address this inherent confound, and further assess the extent to which the reference dependence hypothesis holds when calculations are required. To do this, full subset information was provided within both congruent and incongruent problem presentations, but without reference class totals in either case. By removing these totals, participants could no longer directly select and apply these values as their denominator responses in either congruent or incongruent pairings. This ensured that both types of pairings required the simple computation of adding two subsets, and neither had the potential for interference from an inappropriate reference class total.

In the incongruent problems, eliminating the C+ reference class totals may increase accuracy by removing a value that is hypothesized to interfere with reasoning about the correct nested set (e.g., Reyna, 2004; Reyna and Brainerd, 2008). Though if, as in Experiment 1a, reasoners are drawn to the superordinate value in the response prompt, the hypothesized increase in accuracy may not be as strong as originally hypothesized. If reasoners exhibit a value selection bias, the superordinate set is the only remaining higher order value in the problem text. Thus, removing C+ may shift reasoners to even greater reliance on the overall sample value as their preferred denominator compared to the proportion observed in Experiment 1a.

For congruent pairs, we can examine whether increases in accuracy are the result of a straightforward mapping from problem focus to question asked or because no calculations were required to reach the correct solution. With the T+ reference class removed, the mapping should still be straightforward, but the need to add the two subsets (C+T+ and C–T+) will reduce accuracy if computation is responsible for performance deficits.

Any decrease in accuracy with a need for computation could be the result of one of two different mechanisms. First, accuracy may decrease because the arithmetic step may be completed incorrectly, either resulting in “quasi-Bayesian responses” (i.e., responses that use the correct component values but are not combined correctly; Macchi, 2000) or incorrect values. In this case, there should be an increase in responses that are close to correct but computationally inaccurate. Second, accuracy could decrease due to value selection bias. In this case, there should be an increase in other values from the problem being utilized as the denominator.

For both incongruent and congruent problems, difficulties with computations should be especially apparent for those with low numeracy compared to those with higher numeracy (Schwartz et al., 1997; Reyna and Brainerd, 2008; Chapman and Liu, 2009; Talboy and Schneider, 2017, 2018b). Those who experience more difficulty working with numeric information routinely tend to perform worse on computational reasoning tasks than those with higher numeracy (Lipkus et al., 2001; Schwartz et al., 2005; Peters et al., 2006; Garcia-Retamero and Galesic, 2010; Hill and Brase, 2012; Johnson and Tubau, 2013).

### Method

#### Participants

Data from an additional 118 Experiment 1 participants were evaluated here, comprising two between-subjects conditions with either congruent or incongruent full subset problems without reference class totals. These data were compared to the findings from the two conditions in Experiment 1a that presented full subset information with reference class totals (for 236 participants total).

#### Design

This experiment employed a 2 × 2 Congruence (congruent, incongruent) × Reference Class Totals (included, omitted) between-subjects design. Information about reference class totals was either provided (in the congruent and incongruent full information conditions from Experiment 1a) or omitted (in the congruent and incongruent conditions with no reference class totals). An example of how these problems appeared is shown in Table 4 for conditions with reference class totals and in Table 5 for conditions without reference class totals.

**TABLE 5:** Example presentations without reference class totals for the mammography problem.

Incongruent condition-Focus problem without reference class totals
To determine whether a woman is at risk of breast cancer, doctors conduct mammogram screenings. Sometimes women test positive even when they should test negative or test negative when they should test positive Here is some information for a random sample of 10,000 women who had a mammogram:
In this sample of 10,000 women:
Of those who have breast cancer:
80 received a positive result on their mammogram.
20 received a negative result on their mammogram.
Of those who do not have breast cancer:
990 received a positive result on their mammogram.
8,910 received a negative result on their mammogram.
Imagine another random sample of 10,000 women who had a mammogram.

**Congruent test-Focus problem without reference class totals**

To determine whether a woman is at risk of breast cancer, doctors conduct mammograms screenings. Sometimes women test positive even when they should test negative or test negative when they should test positive. Here is some information for a random sample of 10,000 women who had a mammogram:
In this sample of 10,000 women:
Of those who received a positive result on their mammogram:
80 have breast cancer.
990 do not have breast cancer.
Of those who received a negative result on their mammogram:
20 have breast cancer.
8,910 do not have breast cancer.
Imagine another random sample of 10,000 women who had a mammogram.

### Results

A 2 × 2 Congruence (congruent, incongruent) × Reference Class Totals (included, omitted) analysis of covariance was conducted while controlling for numeracy (with corroboration by GEE repeated measures binary regression).

#### Numeracy

As seen in previous problem variations, numeracy significantly predicted response accuracy, *F*(1,231) = 70.90, *p* < 0.001, $\eta_{\text{p}}^{2}$ = 0.24 [Wald *C*^2^(1, *N* = 236) = 55.98, *p* < 0.001, OR = 1.64; 95% CI: 1.44, 1.87]. As expected, stronger numerical skills were related to higher levels of accuracy, *r*_*s*_(234) = 0.48, *p* < 0.001.

#### Congruence and Reference Class Totals

The main effect of congruence on accuracy was not significant, *F* < 1 [Wald *C*^2^(1, *N* = 236) = 1.21, *ns*]. On average, accuracy was virtually the same, and relatively low, for participants viewing congruent (46%; *M*_*adj*_ = 3.64, *SD* = 2.81) versus incongruent problem-question pairings (44%; *M*_*adj*_ = 3.53, *SD* = 2.81).

Additionally, the effect of reference class totals only approached significance, *F*(1,231) = 3.12, *p* = 0.08, $\eta_{\text{p}}^{2}$ = 0.01 [Wald *C*^2^ (1, *N* = 236) = 3.12, *p* = 0.08, OR = 0.69; 95% CI: 0.45, 1.04]. If anything, mean accuracy was slightly lower when reference class totals were provided (41%; *M*_*adj*_ = 3.27, *SD* = 2.79) than omitted (49%; *M*_*adj*_ = 3.91, *SD* = 2.79). This is not entirely surprising as removal of the reference class totals from the incongruent pairings was expected to increase accuracy in incongruent pairings but decrease accuracy in congruent pairings.

**FIGURE 3:**
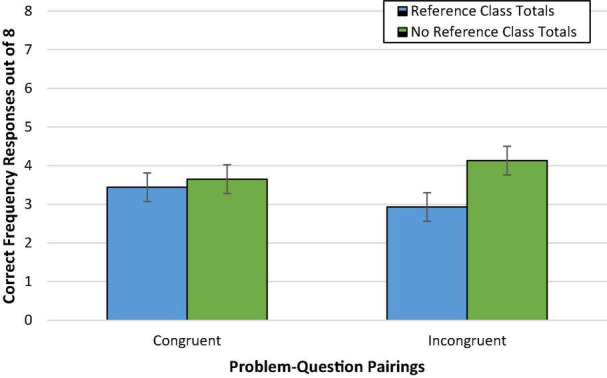
Experiment 1b condition accuracy means while controlling for numeracy. Error bars represent ± 1 SE.

Pivotal to our hypotheses, however, was the Congruence × Reference Class Total interaction effect. As shown in Figure 3, however, this effect was not clearly observed, *F*(1,231) = 2.18, *p* = 0.14 [Wald *C*^2^ (1, *N* = 236) = 2.77, *p* = 0.10]. Exploratory *post hoc* Bonferroni tests did not reveal any potential differences in performance across conditions.

The results suggest that reasoners were not hampered by the need to compute, as roughly the same proportion determined the correct denominator regardless of whether the value was provided directly in the test-focus full information condition (Table 4) or had to be calculated from the subset information in the test-focus condition with no reference class totals (Table 5; and the two condition-focus problems). The lack of a reduction in accuracy when computations were required seems contrary to findings that, all else equal (including numeracy levels), reasoners perform worse on problems that require calculation (e.g., Ayal and Beyth-Marom, 2014; McDowell and Jacobs, 2017). Nevertheless, accuracy was much lower than that documented in previous research (Talboy and Schneider, 2018a,b).

#### Denominator Response Errors

**FIGURE 4:**
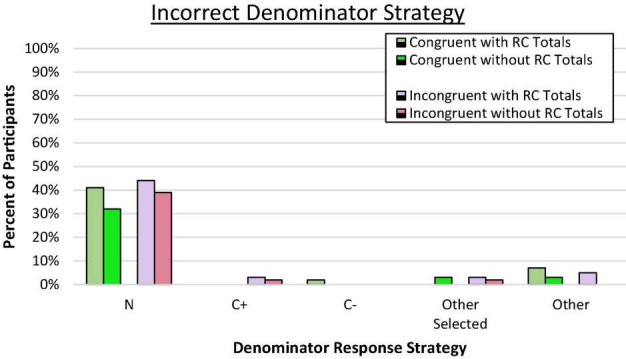
Proportion of participants who consistently used an incorrect denominator strategy on the frequency response format. N denotes the total in the superordinate set (i.e., sample size), C± denotes the total number of people who have or do not have the condition.

We again assessed reference class choice by examining patterns of denominator responses to assess value selection bias and the pattern of errors. In both congruent and incongruent pairings, across all conditions, a large majority of incorrect responses aligned with the superordinate set (*N*) as shown in Figure 4 (*p* < 0.001 by binomial *z* in each condition). Again, the tendency to select the overall sample size rather than any other reference value was similar across conditions, *C*^2^(3, *N* = 236) = 1.85, *ns*. As predicted by the value selection bias hypothesis, reasoners seemed to latch on to the only remaining reference value provided, which was the superordinate value. As shown previously in Experiment 1a, when reference class totals were provided, very few reasoners consistently used the C+ reference class, even when it was presumably salient in the incongruent condition. This same pattern was observed when no reference class values were provided. Reasoners from the two new conditions in this experiment also appeared to be selecting the overall sample size as the presumed reference class of interest.

#### Numeracy and Calculations

We predicted that the performance advantage for those high in numeracy would be particularly strong for congruent problems that required calculation compared to problems not needing computations. The relationship between numeracy and accuracy was fairly strong on congruent problems that required adding two values together to determine the correct denominator, *r*_*s*_(57) = 0.51, *p* < 0.001, and seemed possibly not as important when correct performance did not require calculation, *r*_*s*_(57) = 0.28, *p* = 0.03. Although in the predicted direction, the difference in correlation coefficients was not significant, *z* = 1.42, *p* = 0.08. Thus, there is insufficient evidence that numeracy was more important when calculations were required.

### Discussion

In Experiment 1b, we replicated the relationship between numeracy and accuracy, showing that the relationship was relatively strong even when problems did not require calculation. We again found evidence of value selection bias, with incorrect responses from both congruent and incongruent pairings virtually always corresponding to values provided in the problem rather than computation errors. However, the hypothesis that congruence effects would generalize to conditions requiring calculations was not supported in this study; performance was generally similar across conditions.

We again observed what appears to be a reference dependence effect caused by the request to consider a new sample. As in Experiment 1a, the majority of reasoners who did not determine the correct response consistently utilized the superordinate set (N) as the reference class denominator rather than selecting T+ when available or adding to find the T+ reference class. The salience of the superordinate value was likely enhanced by the new sample prompt immediately preceding response solicitation.

## Experiment 1c: Reference Class Configuration

Experiment 1c assessed the role of reference dependence in diagnostic reasoning by altering the overarching reference class configuration. This was done by comparing performance on problems (1) with the four subsets nested separately into two related reference classes (C+ and C– for incongruent versus T+ and T– for congruent), (2) nested into a single superordinate set (N), or (3) listed on their own with no explicit organizing information (i.e., an unlabeled nesting). By directly manipulating the configuration of the subsets into different types of nested structures, we aimed to further assess the extent to which the presence or type of key reference class referents affects reasoners’ abilities to determine the PPV.

### Reference Class Configuration

Reference dependence suggests that reasoners will rely on the context in which the subsets are organized to determine the solution to Bayesian reasoning problems. In the problem formulations from Experiments 1a and 1b, a visual structuring (indentation) demonstrated how the subsets were nested within or conditional on each of the indicated reference classes. There was a verbal organization describing which subsets belonged to which reference classes, along with information that all of the values were drawn from a larger superordinate set.

In Experiment 1c, we explicitly tested the influence of drawing attention to the potential relevance of the superordinate value by introducing a problem formulation in which the visual organization created nesting under the superordinate set rather than the condition or test reference classes. An example of superordinate set nesting is provided in Table 6. By visually nesting all four possible subsets into the superordinate set, the superordinate becomes an even more explicit reference class on which the subsets are (in effect) conditioned.

**TABLE 6:** Example presentations with superordinate set organization for the mammography problem.

Incongruent condition-Focus problem with superordinate set organization
To determine whether a woman is at risk of breast cancer, doctors conduct mammogram screenings. Sometimes women test positive even when they should test negative or test negative when they should test positive Here is some information for a random sample of 10,000 women who had a mammogram:
Of the 10,000 women in this sample,
80 have breast cancer and received a positive result on their mammogram.
20 have breast cancer and received a negative result on their mammogram.
990 do not have breast cancer and received a positive result on their
mammogram.
8,910 do not have breast cancer and received a negative result on their
mammogram.
Imagine another random sample of 10,000 women who had a mammogram.

**Congruent test-Focus problem with superordinate set organization**

To determine whether a woman is at risk of breast cancer, doctors conduct mammograms screenings. Sometimes women test positive even when they should test negative or test negative when they should test positive. Here is some information for a random sample of 10,000 women who had a mammogram:
Of the 10,000 women in this sample,
80 received a positive result on their mammogram and have breast cancer.
990 received a positive result on their mammogram and do not have breast
cancer.
20 received a negative result on their mammogram and have breast cancer.
8,910 received a negative result on their mammogram and do not have
breast cancer.
Imagine another random sample of 10,000 women who had a mammogram.

If reference dependence plays a primary role in how people go about solving these problems, this superordinate reference point should function similarly to the condition-focus reference class in the standard incongruent problem-question pairing from previous research (e.g., Talboy and Schneider, 2018a,b). Although this already appeared to be a focal reference point in the previous two studies, we expected the proportion who use this value to be even higher in a condition that explicitly organizes subsets within the superordinate set.

In this superordinate nesting, the conditioning on the C or T reference class had to be removed and was replaced by the conjunction of the condition and test status. Thus, the congruence manipulation had to be altered such that congruence or incongruence was reflected in the order of the four subsets and the two premises in each conjunction. Although no longer nested within a condition or test reference class, the order still communicates through primacy an emphasis on the condition (incongruent) or the test. The condition was always indicated prior to the test result in incongruent conditions, and test results were listed first in congruent conditions. This provided an embedded structure that reasoners might include as a reference point for their deliberations, although admittedly the manipulation was by necessity much more subtle.

This congruent ordering of the problem may become more important when all explicit reference points are eliminated from the problem presentation, as they were in the second novel problem formulation created for this experiment. This formulation removed all explicit configural and numeric reference cues such as the visual nesting or presence of reference class or superordinate set values, listing each subset as a condition-test or test-condition conjunction. By removing the structural organization, our intention was to eliminate the reference class cues that intuitively led reasoners to rely on these values for solution.

In this problem formulation, shown in Table 7, there were no visual or verbal nesting components that provided subset-set information about how the groups were related to one another. Although there was still a response prompt to imagine another random sample, there was no numeric value on which to anchor. This was expected to provide insight into what draws reasoners’ attention when there are no explicit cues about the value for the reference class of interest. Nevertheless, congruent pairings were still predicted to produce at least slightly higher accuracy than incongruent pairings because the congruent ordering could be more readily matched to what was being asked for in the problem. This “no organization” manipulation also provided a particularly stringent test of the pervasiveness of the value selection bias because identifying any reference class would require some computation.

**TABLE 7:** Example presentations with no explicit organization for the mammography problem.

Incongruent condition-Focus problem with no explicit organization
To determine whether a woman is at risk of breast cancer, doctors conduct mammogram screenings. Sometimes women test positive even when they should test negative or test negative when they should test positive Here is some information for a random sample of 10,000 women who had a mammogram:
80 have breast cancer and received a positive result on their mammogram.
20 have breast cancer and received a negative result on their mammogram.
990 do not have breast cancer and received a positive result on their
mammogram.
8,910 do not have breast cancer and received a negative result on their
mammogram.
Imagine another random sample of the same number of women who had a mammogram.

**Congruent test-Focus problem with no explicit organization**

To determine whether a woman is at risk of breast cancer, doctors conduct mammograms screenings. Sometimes women test positive even when they should test negative or test negative when they should test positive. Here is some information for a random sample of 10,000 women who had a mammogram:
80 received a positive result on their mammogram and have breast cancer.
990 received a positive result on their mammogram and do not have breast
cancer.
20 received a negative result on their mammogram and have breast cancer.
8,910 received a negative result on their mammogram and do not have
breast cancer.
Imagine another random sample of the same number of women who had a mammogram.

### Method

#### Participants

Experiment 1c consisted of 6 between-subjects conditions, including four novel conditions with 235 of the randomly assigned Experiment 1 participants, plus the two conditions previously introduced in Experiment 1b (353 participants total).

#### Design

This experiment employed a 2 × 3 Congruence (congruent, incongruent) × Organization (reference classes, superordinate set, none) between-subjects design. Each problem variation was presented in incongruent condition-focus or congruent test-focus format, and all versions included full subset information with no condition or test reference class totals in either incongruent or congruent format. Thus, all participants were required to complete computations to determine the correct denominator reference values. Problems were organized in one of three ways: subsets visually nested in condition or test *reference class*es without reference class totals (from Experiment 1b), with all four subsets (C+T+, C+T–, C–T+, C–T–) nested together within the *superordinate* set, or as a listing of the four subsets with no explicit nesting (*none*). The procedure was identical to Experiments 1a and 1b.

### Results

A 2 × 3 Congruence × Organization (reference class, superordinate, none) analysis of covariance was used to analyze the effects of congruence and problem organization on accuracy while controlling for numeracy.

#### Numeracy

There was a strong positive correlation between numeracy and frequency response accuracy, *r*_*s*_(351) = 0.43, *p* < 0.001, suggesting the general numeracy hypothesis is robust across a wide variety of conditions. As a covariate, numeracy significantly predicted response accuracy, *F*(1,346) = 84.51, *p* < 0.001, $\eta_{\text{p}}^{2}$ = 0.20 [Wald *C*^2^(1, *N* = 353) = 62.38, *p* < 0.001, OR = 1.48; 95% CI: 1.35, 1.64].

#### Congruence

**FIGURE 5:**
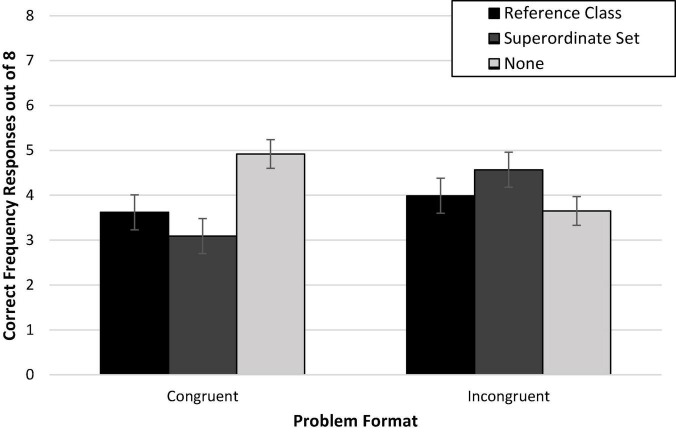
Experiment 1c condition accuracy means while controlling for numeracy. Error bars indicate ± 1 SE.

Against expectations, there was no main effect of congruence on accuracy, *F* < 1 [Wald *C*^2^ (1, *N* = 353) = 0.41, *p* = 0.53]. On average, those who read congruent problem-question pairings (49%; *M*_*adj*_ = 3.89, *SD* = 2.71) performed similarly to those who read incongruent problem-question pairings (51%; *M*_*adj*_ = 4.09, *SD* = 2.69). Both groups solved about half of the problems experienced on average. This may be due in part to the weaker congruence manipulation which is a result of removing the nested problem structure. There was no main effect of problem organization, *F*(2,346) = 1.37, *p* = 0.25 [Wald *C*^2^ (2, *N* = 353) = 2.40, *ns*], but there was a Congruence × Organization interaction, *F*(2,346) = 7.47, *p* = 0.001, $\eta_{\text{p}}^{2}$ = 0.04 [Wald *C*^2^ (2, *N* = 353) = 14.46, *p* < 0.001], which is shown in Figure 5.

Simple effects analysis was completed to evaluate the effect of problem organization within each level of congruence. For those who read congruent problem-question pairings, we expected that accuracy would be highest when nested within the most relevant reference class and would decrease when organizational structure was reduced, especially with the distraction of the explicit superordinate nesting.

Within the congruent pairings (see left side of Figure 5), accuracy was significantly different across the three problem organizations, but was not consistent with the predicted pattern, *F*(2,346) = 7.23, *p* = 0.001, $\eta_{\text{p}}^{2}$ = 0.04 [Wald *C*^2^(2, *N* = 176) = 13.28, *p* = 0.001]. Accuracy was comparable between the presumably helpful reference class structure and the presumably less helpful superordinate organization, *p* = 0.24. However, removal of all explicit organization, including the misleading superordinate value, actually increased accuracy compared to both the reference class organization, *p* = 0.01 (OR = 2.05; 95% CI: 1.14, 3.71), and the superordinate set organization, *p* < 0.001 (OR = 2.74; 95% CI: 1.57, 4.80).

Though surprising, this provides insights into what limits the value of a congruent problem structure. Nesting problem information in terms of the correct reference class does not appear to be especially helpful when that class total is not provided. Nor is ordering of the test premise before the condition premise helpful when a misleading superordinate reference class is present. Instead, what appears to be most helpful is a combination of a congruent structure with enumeration of the reference class that is required for solution, in addition to the removal of misleading superordinate values.

Within the incongruent pairings (see right side of Figure 5), accuracy did not differ as a function of problem organization, *F*(2,346) = 1.71, *p* = 0.18. Accuracy on these incongruent pairings did not appear to change as the problem structure was altered from the misleading condition reference class nesting to another misleading reference class (i.e., the superordinate value) or to no explicit reference point. This suggests that reasoners were not helped, nor necessarily hindered further, by changes in the explicit organization when there was nothing in the problem to help them pick out the relevant reference point.

*Post hoc* Bonferroni comparisons showed the only other effect in this experiment was an unexpected disadvantage of congruent relative to incongruent pairings that used the superordinate organization (*p* = 0.04, *d* = –0.55; OR = 0.43; 95% CI: 0.25, 0.74). Additional future testing will be needed to corroborate this potential reversal when congruence is only represented as subset ordering.

#### Error Response Patterns

**FIGURE 6:**
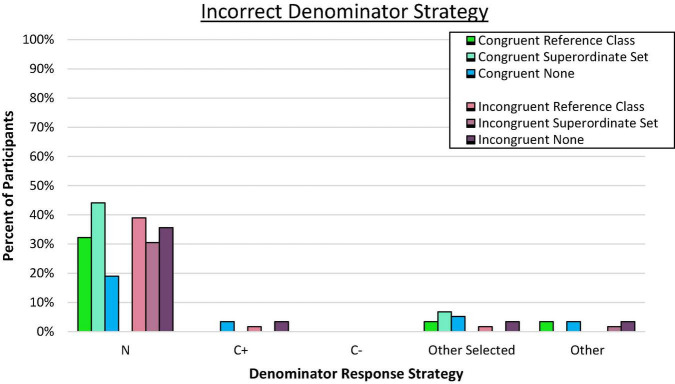
Proportion of participants who consistently used the incorrect denominator strategy on the frequency response format. N denotes the total in the superordinate set (i.e., sample size), C± denotes the total number of people who have or do not have the condition.

Denominator errors (given on at least four of the eight problems) were coded using the same process as in Experiments 1a and 1b, and are shown in Figure 6. Based on the previously observed salience of the superordinate value within the problem and response prompt, we expected that the dominant error would be the total sample size in all conditions except for the No Explicit Organization condition. This is the only condition in which the superordinate value was not explicitly provided.

As expected, a large majority of error responses involved selecting N as the denominator in the Reference Class and Superordinate Set conditions (*p* < 0.001 by binomial *z* in each condition), but the use of N was also dominant in the Incongruent No Explicit Organization condition (binomial *z* = 2.89, *p* < 0.01) even though that value was not present in the problem. The superordinate value was also the most common type of denominator error in the Congruent No Explicit Organization condition, but it did not represent a significant majority of all error responses (binomial *z* = 0.94, *ns*). Nevertheless, the general bias toward using the superordinate over other options for the reference class was not markedly different across conditions as a whole, *C*^2^(5, *N* = 353) = 9.66, *p* = 0.22.

The superordinate response error was most surprising in the No Explicit Organization conditions. Reasoners who evaluated problems in this format were not given numeric totals and would have to perform a calculation to generate any possible reference class. Generating the C+ or T+ total required summing across two groups, whereas determining the total sample required summing all four subsets. If computing ease were a primary consideration as suggested in previous research (Johnson and Tubau, 2015, 2017; Talboy and Schneider, 2018a,b), this organizational structure should have reduced the tendency to rely on the superordinate value because it requires more mathematical manipulation (although still quite simple by most standards) compared to other potential reference classes of interest. Performing the addition calculation was apparently not an obstacle as 19 and 36% (congruent and incongruent, respectively) of participants consistently used this value as their preferred denominator.

In the no organization condition, they had no superordinate or other organizing information except for either the congruent test-condition ordering of groups or the incongruent condition-test ordering, and a response prompt that directed attention back to a new sample of the same size. Although no sample size value was provided, at least some participants may have (erroneously) inferred that the prompt to consider a new sample required that the entire sample be adopted as the reference class. This seems especially likely given that, throughout, the most common error across all problem format variations was the superordinate *N* value.

In the incongruent no organization condition, it seems the condition-test subset order did not draw added attention to the possibility of C+ as the relevant denominator (i.e., the typical error expected in incongruent problem organizations). However, the congruent test-condition order may have helped some to see that only the two test-positive groups were relevant, and as a result, fewer relied on the superordinate total as their denominator than the incongruent version, *C*^2^(1, *N* = 117) = 4.54, *p* = 0.03, Cramer’s *V* = 0.20, even though this same congruent ordering seemed to have no facilitative effect, or potentially a reversal, compared to the incongruent order when nested within the superordinate set, *C*^2^(1, *N* = 118) = 2.32, *p* = 0.13.

It is important to note that in both of the no organization conditions at least 25% of participants (and as many as 42%) computed a value to submit as their denominator rather than selecting a value from the problem. This is the only finding that goes against the value selection bias, and instead suggests that there is a limit to the tendency of reasoners to simply opt to use values given in the problem.

If no candidate values within the problem seem to have the potential to be the value needed, reasoners will compute (at least through simple addition) values that seem to have more potential to be what is required. In this case, participants seem to recognize that this problem structure requires that the denominator be a number that represents a reference class that takes a larger group into account. This suggests that participants do, almost unanimously, recognize the need to identify a larger reference class for the denominator, but that they may be confused by which one is required.

### Discussion

In Experiment 1c, we found partial support for the importance of the structural organization of the problem, however, this was not always facilitated by the congruence manipulation. The congruence hypothesis was only supported in Experiment 1c when all explicit organizational cues were eliminated from the problem presentation, but not on problems that organized subsets into the superordinate set, where the pattern potentially reversed, or when organized within reference class but without enumerated totals.

When reasoners were not correctly calculating the denominator value, most utilized the superordinate value (N). It appears that the superordinate value (N) was a salient reference point in the problem organizations, even when the value for N was not explicitly stated. Instead of demonstrating a value selection bias in this case, reasoners were calculating the total N from the four subsets. Numerical skill was again a strong predictor of accuracy across all conditions.

## Experiment 2: New Sample Value as Reference Point

Thus far, the reason behind the widespread adoption of the overall sample size as reference point has been assumed to be its proximal introduction when asking for estimates from a new sample of the same size. Re-introducing the sample size makes this value salient. Based on a value selection bias, the re-introduction of this numeric reference would make the value more likely to be adopted as the relevant reference point. In Experiment 2, we directly test this possibility by collecting additional data in a one-way experimental design comparing responses to the original version of the congruent problems used in Talboy and Schneider (2017, 2018a) to each of the format updates introduced to create the congruent stimuli in Experiment 1. The four updates to the original problem included:

(a) New Sample – introducing a new superordinate sample into the PPV question,
(b) Non-redundant – removing the reiteration of the desired reference class in the PPV question,
(c) No Bold – removing bold throughout the problem, and
(d) Paper – supplying participants with paper to assist in calculations.

If introducing the instruction to imagine a new sample makes the sample size loom larger as a potential reference point, we expected to see more errors on the PPV question when the new sample was introduced compared to the original version of the problems which did not mention a new sample. In particular, we expected to see more widespread adoption of the superordinate as denominator when the exact size of the set is made salient by reinforcing the superordinate value. We also wanted to see whether any of the other potential assists, such as bolding of critical problem elements, repeating critical reference points in the PPV question, or providing paper for notes and calculations is likely to aid in problem solution.

### Method

#### Participants

Experiment 2 was conducted with a sample of 364 randomly assigned psychology undergraduates (net after removing data from 3 participants who failed to complete the study; *n* = 72–75 per condition), awarded course credit for participating. Power analysis confirmed just over 80% power for intermediate effects (*f* = 0.25) in pairwise comparisons and 95% power in combined tests, with similar or slightly greater power for binomial comparisons.

#### Design

This experiment employed a one-way between-subjects design varying aspects of the presentation of the diagnostic problem. All of the conditions used the partial-subsets (standard) version of the congruent problems as described in Experiment 1a, with slight modifications.

The *original* condition presented problems just as they were presented in Talboy and Schneider (2017, 2018a), which are similar to the format shown in Table 1 but with bolding of critical values, phrasing of the PPV question with a redundant reminder of the reference class (e.g., “Based on the number of women who would test positive, how many of these women who test positive would you expect to actually have breast cancer?”), no paper available, and no mention of a new sample.

The *new sample* condition looked like the original condition, but with no bold and the question format altered to read, e.g., “Now, imagine another representative sample of 10,000 women who had a mammogram. Based on the number of women from this new sample who would test positive, how many of these women who test positive would you expect to actually have breast cancer?”

The *non-redundant* condition looked just like the original, but in this case the PPV question was simplified to read, e.g., “Of the women who test positive, how many do you expect to have breast cancer?”

The *no bold* condition looked just like the original except that there was no bolding anywhere in the problem.

The *paper* condition used the original problem version, but participants were given a two-sided sheet of paper with each side separated into quadrants numbered 1–8. Participants were instructed at the outset to place any notes or calculations they might want to make in the quadrant matching the current problem. Similar to the other conditions in the previous experiments, no calculators were permitted.

As in the previous experiments, the dependent variable in all conditions was average accuracy on the frequency response format for the eight diagnostic reasoning problems (range: 0–8 correct responses).

The procedure was otherwise identical to Experiment 1.

### Results

A one-way analysis of covariance was used to analyze the effects of problem presentation on accuracy while controlling for numeracy (corroborated by repeated measures binary logistic regression).

#### Numeracy

As expected, numeracy (*M* = 4.48, *SD* = 1.87, Cronbach’s a = 0.67, Min = 0, Max = 8) was significantly related to PPV performance, *F*(1,358) = 84.16, *p* < 0.001, $\eta_{\text{p}}^{2}$ = 0.19 {Wald *C*^2^ (1, *N* = 364) = 78.53, *p* < 0.001, OR = 1.66, 95% CI [1.48–1.85]}, with *r*_*s*_(362) = 0.43, *p* < 0.001. This is especially noteworthy as none of the congruent problems used in Experiment 2 required computation to find the T+ reference class. Confirming the findings of Experiment 1b, those who were more adept at working with numbers tended to perform better in this task even in cases where computation was not required.

#### Problem Presentation

**FIGURE 7:**
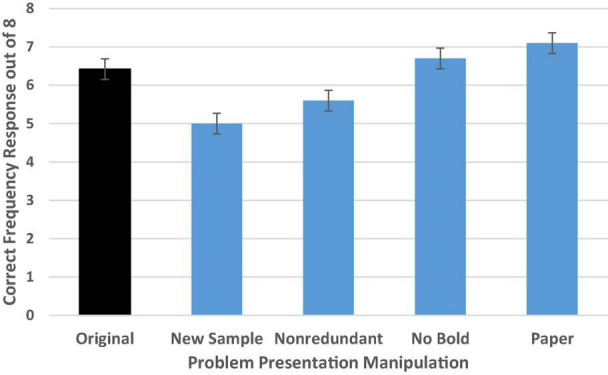
Experiment 2 condition accuracy means while controlling for numeracy. Error bars indicate ± 1 SE.

As shown in Figure 7, there was a moderately large general effect of problem presentation differences on PPV identification, *F*(4,358) = 9.50, *p* < 0.001, $\eta_{\text{p}}^{2}$ = 0.10 [Wald *C*^2^ (4, *N* = 364) = 35.83, *p* < 0.001]. We replicated the findings from Talboy and Schneider (2018a,b), with reasoners demonstrating impressive performance on the original problem format with an adjusted average of 6.42 correct out of 8 (*SD* = 2.31) or 80% accuracy.

Simple contrasts comparing performance in each condition to those who read the original problem format confirmed our suspicion. Accuracy deteriorated when a new sample was introduced, with average performance dropping to 5.0 out of 8 (*SD*’s = 2.31) or 63% accuracy (*p* = 0.001, *d* = 0.60; OR = 0.38 CI [0.20–0.72]). This provides additional evidence that the new sample may have confused or distracted participants in their attempt to isolate the needed reference class.

In addition, we found that accuracy in the non-redundant condition was slightly lower than in the original problem format (*p* = 0.04, *d* = 0.31; OR = 0.50 CI [0.26–0.96]). This demonstrates that the reiteration of the test positive reference class in the wording of the PPV question in the original problem format may have helped clarify which reference class was the one of interest (see also, Galesic et al., 2009). There was not a significant difference in accuracy rate for either the no bold or the paper condition compared to the original (bolded, paperless) format.

Additional follow-up tests with Bonferroni correction confirmed that performance in the new sample condition was significantly worse than performance in either the no bold (*p* < 0.001, *d* = 0.70) or paper conditions (*p* < 0.001, *d* = 0.89) but was not clearly different from performance in the non-redundant condition. Performance in the non-redundant condition was significantly worse than in the paper condition (*p* = 0.001, *d* = 0.65), and approached significance in the comparison with performance in the no bold condition (*p* = 0.06, *d* = 0.46). No other tests were significant, suggesting that neither bolding critical information in the problem nor providing paper (at least when computation is not required) provides meaningful assistance in helping to find the needed reference class to identify the PPV.

#### Error Response Patterns

**FIGURE 8:**
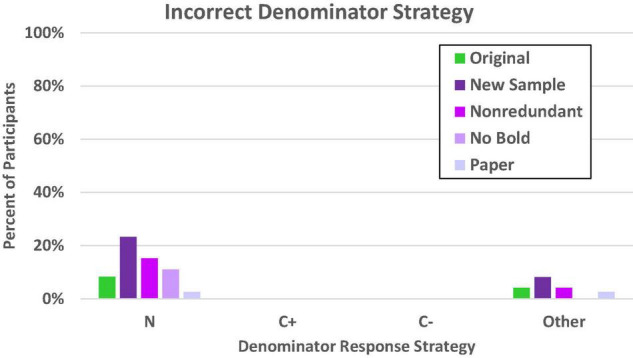
Proportion of participants who consistently used the incorrect denominator strategy on the frequency response format. N denotes the total in the superordinate set (i.e., sample size), C± denotes the total number of people who have or do not have the condition, other includes both selected and not readily identifiable values.

We again evaluated denominator errors (given on at least four of the eight problems) as in Experiments 1a–1c. These are shown in Figure 8. As in the previous study, the most common error in general was to rely on the total sample size (N) rather than any other value provided in the problem. Nevertheless, it is also obvious that there are large differences in reliance on this value for the reference class, *c*^2^(df = 4, *N* = 364) = 16.59, *p* = 0.002. As expected, the participants who were most tempted by the superordinate value were those for whom the superordinate value had been made especially salient by the introduction of a new sample. This is consistent with what would be expected with a value selection bias, though the pattern is not as conspicuous here (23%) as in the comparable (congruent) conditions in Experiment 1a (approximately 40%).

Although it appears there might be a slightly higher error rate for the new sample condition in the selection of values that did not correspond to the N, C+, or C– reference classes, the general trend across the conditions was not significant, *c*^2^(df = 4, *N* = 364) = 6.98, *p* = 0.14. There was no tendency whatsoever for any of the five test-focus groups to be tempted to use either the C+ or C– reference class as an anchor, which is consistent with previous findings that using a congruent problem format helps reduce and even eliminate confusion between the T+ and C+ (or C–) reference classes.

### Discussion

In Experiment 2, we documented that the reinforcement of a new sample with a salient superordinate value tempts participants to adopt that value, rather than evaluating more carefully to recognize that T+ is the correct reference class. This is consistent with the hypothesis of a value selection bias. Moreover, the congruence hypothesis was supported in that all of the conditions, with the possible exception of the new sample condition, showed high accuracy levels similar to those observed in Talboy and Schneider (2018a,b).

Another finding that is relevant to reference class is the tendency for performance to drop when the needed reference class is not reiterated as part of the PPV question. In the non-redundant condition, the reference value of interest was not reinforced, suggesting clarity in both the problem format AND the question facilitate correct solution. Omitting this redundancy in Experiment 1, along with including a salient reminder of the superordinate sample, seems to account for the drop in the ability of congruence to help steer participants in the direction of the correct PPV.

## General Discussion

The goal of these experiments was to evaluate the representational and computational aspects of simplified Bayesian reasoning tasks as they relate to reference dependence. The most consistent finding in the first experiment was that people were much more likely than expected to utilize the superordinate value as part of their solution, regardless of organizational structure. This resulted in a weakened effect of congruence, with relatively low accuracy even in congruent conditions, as well as a different pattern of response errors than what was originally anticipated.

There was consistent and strong evidence of a value selection bias in that incorrect responses almost always conformed to values that were provided in the problem rather than errors related to computation. The one notable exception occurred when no organizing information was available in the problem, other than the instruction to consider a sample of the same size as that in the problem. In that case, participants were most apt to sum all subsets of the sample to yield the original sample size (N).

In Experiment 2, we showed that the most likely cause of the reduced facilitative effect of congruence was the instruction to consider a new sample. Although congruence helps draw attention to the needed reference class, referring to a new sample of a specified size seems to largely undo this effect, drawing attention away from the correct reference class to the alternative but incorrect superordinate set.

This suggests that naïve problem solvers are highly susceptible to cues that might indicate which reference class is relevant. This, in turn, supports the larger reference dependence hypothesis by suggesting that one of the fundamental difficulties in Bayesian reasoning tasks and other diagnostic reasoning problems is the inability to readily isolate the correct reference class. The suggestion that redundancy helps when mentioning the needed reference class within the PPV question also supports this possibility. Latching on to salient values provided within the problem also indicates a lack of conviction about what is being asked or what is needed to produce the correct response. This susceptibility to given reference points may contribute to the relational alignment problem identified by Johnson and Tubau (2017) and Tubau et al. (2019).

In both our experiments, higher numerical skills were generally associated with higher accuracy, whether calculations were required or not. This may extend appreciation to the role of numeracy not just in computation but also in analysis of what is being asked in numeric reasoning problems.

### Reference Dependence

Like many other types of reasoning, we provide evidence that reference dependence may be a crucial aspect in Bayesian and other forms of diagnostic reasoning. The results of these experiments indicate that the initial presentation of the problem directly informed how reasoners responded to the PPV question. Although the nesting of problem subsets was predicted to be the primary source used by problem solvers to select the relevant reference class, we found unexpectedly that the request to consider a new sample just prior to generating a solution pulled attention to a different reference point: the superordinate value (N).

Many previous studies have reported similar accuracy rates regardless of whether required PPV estimates referred to the initial sample (e.g., Sirota et al., 2014a) or to a new representative sample of the same size (e.g., Gigerenzer and Hoffrage, 1995). A large-scale meta-analysis evaluated problem forms that explicitly stated a superordinate value in the problem description (but not necessarily as a request to consider a new sample of the same size) and found that having this information available did not affect performance (McDowell and Jacobs, 2017).

In many of the studies in the meta-analysis, accuracy on problems was consistently low, perhaps masking any new sample effect. Recently, Johnson and Tubau (2017) reported a decline in performance when a new sample was introduced, revealing as we did the tendency to opt for N as the denominator or reference class. They observed this effect with and without a numeric value associated with the new sample, which is consistent with our observation of reliance on the overall sample even in a condition referencing a same-sized new sample without a numeric referent. This reliance on the superordinate set when asked to consider a new sample lends support to the larger hypothesis that reference dependence plays a vital role in performance on Bayesian reasoning tasks. Even in the simplest congruent problem-question pairings tested in Experiment 1a, wherein the correct denominator value could be directly selected from the problem, the focus on the superordinate value provided enough pull that accuracy was only around 50% rather than at the expected 80% mark.

This reduction in accuracy was reversed in Experiment 2 by leaving out the instruction to consider a new sample, thereby returning accuracy rates to 80% or more. This demonstrates both the facilitative effects of the congruent problem presentation with its emphasis on the correct T+ reference class, as well as the debilitating effects of introducing a different possible referent immediately prior to being asked the PPV question.

### Congruent Problem Structuring

The congruent format was expected to increase accuracy because the reference values highlighted in the problem structure through verbal and visual cues aligned with the question of interest (Talboy and Schneider, 2018a,b). This prediction was borne out in Experiment 2, wherein accuracy rates were 80% or higher in all congruent conditions except for the New Sample condition.

In Experiment 1, however, the effect of congruence was weak compared to previous research (e.g., Talboy and Schneider, 2018a,b). Further, the congruence effect was not observed at all when the problems were organized without an enumerated reference class or with only a superordinate set. The weak or absent effect of congruence is likely the result of focusing attention on the superordinate (N) value rather than the relevant reference class. This highlights the importance of problem and question structuring, especially signaling the implied reference point for obtaining the solution.

It also reveals the vulnerability of naïve problem solvers in selecting the relevant reference point. Even small changes in problem and question structure can apparently confuse reasoners into selecting an inappropriate reference value for answering questions about diagnostic tests. It is unclear whether they are aware of this vulnerability or if perhaps they feel confident in their answers despite the ease with which a simple wording change can alter their conclusions.

#### Amount of Information

Reasoners create mental representations of the problem structure based almost exclusively on the information that is provided (Kintsch and Greeno, 1985; Johnson-Laird, 1994; Sirota et al., 2014a). Based on the mental models approach, we hypothesized that full subset information would increase accuracy relative to partial information. However, a competing discrimination hypothesis suggested the reverse because reasoners may have greater difficulty discriminating among a larger set of values.

Neither hypothesis was supported, with no discernible effect of partial versus full information on accuracy, regardless of congruence. For both, accuracy on congruent pairings was moderate or low. Accuracy on incongruent pairings was also relatively low as has been found in many previous studies (e.g., Gigerenzer and Hoffrage, 1995; Gigerenzer et al., 2007; Reyna and Brainerd, 2008; Hoffrage et al., 2015; Johnson and Tubau, 2015; Sirota et al., 2015). However, the tendency for the superordinate value (*N*) to become a salient reference point may have overshadowed any effect of full versus partial subset information. A future study could reassess whether full subset information alters accuracy without the distraction of a second salient reference point.

#### Removing Interference From Misleading Reference Points

Changing or removing focal reference values from the problem affected accuracy for reasoners primarily in the congruent pairings. We also observed an unexpected reversal of the effect of congruence. This result is clouded both by the reliance on the superordinate value when a new sample was introduced, and potentially by the change in subset representation from a conditional to a conjunctive format.

Even as a simple manipulation of order of premises in a conjunction, there was some evidence that this manipulation may influence performance. These findings seem worthy of focus in future studies especially given related research on conditional reasoning suggesting that these representations may alter a reasoners’ understanding of conditional rules (see, e.g., Oberauer and Wilhelm, 2003; Kleiter et al., 2018). These understandings, in turn, may reflect different assumptions about relevant reference values.

### Value Selection Bias

In addition to reference dependence and congruence effects, we investigated the general bias toward selecting values from the problem rather than completing calculations when reasoners were unsure of how to determine the solution. In each experiment, virtually all reasoners who did not determine the correct response utilized values directly from the problem to fill in each component of their solution, except when all values referred to subsets with no feasible value presented that could reasonably be the needed reference class. Hardly any reasoners provided responses that were consistent with calculation errors. This provided strong support for the value selection bias, while also confirming the strong draw to the superordinate set value as the denominator.

When reasoners are not familiar with the problem that has to be solved, they tend to rely on surface features to guide their solution (Winner et al., 1980; Chi et al., 1981a,b; Owen and Sweller, 1989; Swanson and Beebe-Frankenberger, 2004). This tendency may reflect a reluctance to engage the mental resources needed to fully flesh out the problem space (e.g., “the lazy controller,” Kahneman, 2011), which results in responses consistent with the identifiable problem values rather than calculation mistakes. This bias may also reflect a general belief that relevant values should be readily available in the problem description, and so calculations should not be needed (Talboy and Schneider, 2018a). Like the matching bias observed in studies of conditional reasoning (e.g., Evans et al., 2003), we consistently observed this value selection bias as the default response strategy when reasoners did not determine the correct response. This highlights the need to identify and develop methods for overcoming reliance on values in the problem to focus attention instead on what steps are actually needed to get to the correct solution.

### Calculations

Much of the existing literature suggests that computational difficulties make Bayesian reasoning tasks inherently difficult, particularly for those with low numerical skill (Schwartz et al., 1997; Reyna and Brainerd, 2008; Chapman and Liu, 2009; Talboy and Schneider, 2018b). Although some argue that this basic addition operation is simple in frequency format problems (Johnson-Laird et al., 1999; Sloman et al., 2003), there is substantial evidence that many reasoners are unable to complete this simple step to correctly solve the problem (Mayer, 2003; Reyna and Brainerd, 2008; Johnson and Tubau, 2015). However, the difficulty when dealing with simple frequencies is not necessarily with the calculation itself, but with determining the relationship between different subsets and the relevance of different values.

In the simple frequency format, we did not find evidence that an added calculation step interfered with solving the congruent problem (Experiment 1b). Even with no organizing value and a non-numeric prompt to consider a new sample (Experiment 1c), a large portion of reasoners who read congruent (19%) or incongruent (36%) pairings actually calculated the superordinate value. This suggests that reasoners can and do complete simple calculations when they are given cues suggesting these are important values.

The lack of a discernible difference in accuracy in the congruent pairings, regardless of whether calculations were required or not, suggests that a simple addition step is not what is inhibiting accuracy on these nested set problems. This suggests that the breakdown occurs in conceptual or analytic prerequisites for computation (Talboy and Schneider, 2018a) or in the general process of understanding how nested subsets function in relation to one another (see, also Reyna, 2004; Reyna and Brainerd, 2008; Johnson and Tubau, 2017; Tubau et al., 2019).

## Conclusion

What may appear to be simple changes in how reasoning problems are presented can alter the way reasoners interpret and utilize problem information to determine solutions. When reasoners are not sure what they need to determine the correct response, they tend to utilize the values provided directly in the problem presentation. Which value they choose will critically depend on the reference points they identify based on the problem structure and the way the question is asked. Making values salient, for instance, by introducing a new sample, can have a strong influence on responses, misleading reasoners away from the relevant reference point. When calculation is needed, the simple step of summing values does not in itself appear to be a hindrance to accuracy. This again suggests the issue is knowing which values are relevant and how to organize those values cognitively to arrive at the correct solution.

## Data Availability Statement

The raw data supporting the conclusions of this article will be made available by the authors, without undue reservation.

## Ethics Statement

The studies involving human participants were reviewed and approved by University of South Florida IRB. Written informed consent for participation was not required for this study in accordance with the national legislation and the institutional requirements. The participants provided their verbal consent to participate in this study.

## Author Contributions

AT designed, conducted, and evaluated all of the research. SS participated in each step and provided overall guidance throughout the design and reporting phases. Both authors contributed to the article and approved the submitted version.

## Conflict of Interest

AT was employed by the company Microsoft. The remaining author declare that the research was conducted in the absence of any commercial or financial relationships that could be construed as a potential conflict of interest.

## Publisher’s Note

All claims expressed in this article are solely those of the authors and do not necessarily represent those of their affiliated organizations, or those of the publisher, the editors and the reviewers. Any product that may be evaluated in this article, or claim that may be made by its manufacturer, is not guaranteed or endorsed by the publisher.

## Abbreviation

PPV: positive predictive value.
